# *Candida auris* Testing by the Antimicrobial Resistance Laboratory Network, United States, 2022–2023

**DOI:** 10.3201/eid3202.251043

**Published:** 2026-02

**Authors:** Jessica E. Laury, Kaitlin Forsberg, Elizabeth L. Berkow, Sophie Jones, D. Joseph Sexton, Dawn M. Sievert, Shawn R. Lockhart, Jeremy A.W. Gold, Meghan Lyman

**Affiliations:** Centers for Disease Control and Prevention, Atlanta, Georgia, USA

**Keywords:** *Candida auris*, candidemia, fungi, antimicrobial resistance, cross infection, infection control, United States

## Abstract

During 2022–2023, the Antimicrobial Resistance Laboratory Network tested 8,033 *Candida auris* clinical isolates in the United States. Overall, 95% of isolates were fluconazole resistant, 15% amphotericin B resistant, and 1% echinocandin resistant. Laboratory capacity for *C. auris* identification and antifungal susceptibility testing is essential to address this emerging public health threat.

*Candida auris* is an urgent public health threat because of frequent multidrug resistance, high transmissibility in healthcare settings, and association with high-mortality invasive infections (*1*–*5*). The Centers for Disease Control and Prevention Antimicrobial Resistance Laboratory Network (AR Lab Network) adopted *C. auris* testing, including antifungal susceptibility testing, in 2016 to meet clinical and public health needs (https://www.cdc.gov/antimicrobial-resistance-laboratory-networks/php/about/testing-services.html). National annual *C. auris* clinical case counts have increased from <100 in 2016 to >4,500 in 2023 (*5*,*6*). To inform prevention, clinical practice, and surveillance efforts, we describe 2022–2023 AR Lab Network *C. auris* clinical isolate testing.

Clinical *C. auris* isolates are obtained from patient specimens collected during clinical care, not for colonization detection, and can be from any body site (*5*). Isolates were identified by matrix-assisted laser desorption/ionization time-of-flight mass spectrometry. MICs for fluconazole and echinocandins (anidulafungin, micafungin) were determined by using frozen custom broth microdilution panels and, for amphotericin B, by using gradient diffusion strip. According to tentative breakpoints (https://www.cdc.gov/candida-auris/hcp/laboratories/antifungal-susceptibility-testing.html), isolates were considered echinocandin-resistant if resistant to either echinocandin and panresistant if resistant to all 3 antifungal classes.

We examined the number of clinical isolates tested and antifungal susceptibility testing results by year, AR Lab Network region of specimen collection (https://www.cdc.gov/antimicrobial-resistance-laboratory-networks/php/about/domestic.html), and body site. We analyzed clinical specimens only to avoid biases from local screening intensity and protocol differences. We excluded specimens for which it was unclear whether they originated from colonization screening versus clinical isolates (≈14%).

During 2022–2023, a total of 8,033 clinical isolates were tested (Table). Most were from the West (24%), Southeast (21%), or Northeast (19%) regions; <1% were from the Central region. The number of clinical isolates increased from 3,064 in 2022 to 4,969 in 2023, increasing in all regions except the Mountain region (288 to 238). The most common body sites were blood (36%) and urine (32%). The distribution of body sites was similar across regions and years (data not shown).

**Table:** Clinical isolates of *Candida auris*, by region and body site, Antimicrobial Resistance Laboratory Network, United States, 2022–2023

Characteristic	No. (%) isolates		
	All, n = 8,033	2022, n = 3,064	2023, n = 4,969
Region where specimen was collected			
Central	37 (0)	2 (0)	35 (1)
Mid-Atlantic	513 (6)	156 (5)	357 (7)
Midwest	904 (11)	329 (11)	575 (12)
Mountain	526 (7)	288 (9)	238 (5)
Northeast	1,512 (19)	665 (22)	847 (17)
Southeast	1,672 (21)	647 (21)	1,025 (21)
West	1,967 (24)	775 (25)	1,192 (24)
Not reported	902 (11)	202 (7)	700 (14)
Body site			
Blood	2,856 (36)	1,185 (39)	1,671 (34)
Respiratory	715 (9)	277 (9)	438 (9)
Urine	2,598 (32)	933 (30)	1,665 (34)
Wound	1,026 (13)	393 (13)	633 (13)
Other	838 (10)	276 (9)	562 (11)

Overall, 95% (7,244/7,594) of tested isolates were fluconazole-resistant; that percentage exceeded 90% in all regions except the Midwest (83%, 666/801) (Appendix). In total, 15% (1,128/7,458) of isolates were amphotericin B–resistant; that percentage was <5% in all regions except the Central (22%, 4/18), Northeast (44%, 629/1,420), and Mid-Atlantic (62%, 290/469). Fewer isolates were echinocandin-resistant (1%, 97/7,574); the highest percentages were in the Midwest (2%, 13/799), Northeast (2%, 31/1,420), and Mountain (3%, 14/463) regions. Overall, 16/7,438 (<1%) isolates were panresistant, mostly from the Northeast (n = 10).

Fluconazole resistance was higher in 2023 (96%, 4,441/4,616) than in 2022 (94%, 2,803/2,978); the largest differences were in the Midwest (90% [433/481] vs. 73% [233/320]) and Southeast (95% [906/957] vs. 90% [582/646]). Amphotericin B resistance was higher in 2023 (19%, 838/4,497) than in 2022 (10%, 290/2,961); the largest difference was in the Northeast (64% [508/794] vs. 19% [121/626]). Echinocandin resistance was 1% in both years, but in the Mountain region, it was higher in 2023 (7%, 13/187) than in 2022 (<1%, 1/276). Antifungal resistance was similar across body sites for fluconazole. More echinocandin-resistant isolates were from urine (3% [72/2,470] vs. <1% for each other site), and fewer amphotericin B–resistant isolates were from wounds or respiratory sites (10% [89/861] for wounds, 13% [99/761] for respiratory, vs. >15% each other site).

This analysis of AR Lab Network *C. auris* testing revealed a 1.5-fold increase in clinical isolate testing volume from 2022 to 2023, mirroring increases in national *C. auris* case prevalence. The proportion of clinical isolates tested by region generally mirrored regional proportions of national case counts (*6*). Fluconazole resistance rates were slightly higher in 2023 (96%) versus 2022 (94%) and were higher than in 2020 (86%), potentially because of increased circulation of fluconazole-resistant strains, primarily driven by isolates from the Midwest, where the fluconazole resistance rate was 90% in 2023 versus 11% during 2018–2020 (*5*). Amphotericin B resistance rates were higher in 2023 (19%) than in 2022 (10%) but were lower overall during 2022–2023 (15%) compared with 2020 (26%) (*5*). That finding might reflect lack of amphotericin B drug selection pressure, because maintaining resistance likely incurs fitness costs, or changes in circulating strains (*7*).

The frequency of echinocandin resistance (1%) and panresistance (<1%) among *C. auris* isolates remains low, including among blood isolates, supporting use of echinocandins as first-line therapy against *C. auris* infections. However, the number of resistant isolates has increased, and possible spread among patients has been documented (*8*,*9*). Echinocandin resistance was found more often in urine than in blood isolates (3% vs. 1%), which might relate to the limited urinary excretion of echinocandins (*10*).

AR Lab Network *C. auris* testing primarily supports local detection and outbreak response, rather than serving as nationally representative surveillance. Testing performed outside the network, an increasing proportion in recent years, is not captured. Data could not be analyzed at the patient level (including repeat isolates) and lacked information on antifungal exposure, clade, and facility type. Nonetheless, our findings highlight the persistence of *C. auris* as a multidrug-resistant threat requiring sustained investment in laboratory capacity for early detection and response.

Appendix Additional information for *Candida auris* testing by the Antimicrobial Resistance Laboratory Network, United States, 2022–2023.

## References

[1] Lockhart SR, Etienne KA, Vallabhaneni S, Farooqi J, Chowdhary A, Govender NP, et al. Simultaneous emergence of multidrug-resistant *Candida auris* on 3 continents confirmed by whole-genome sequencing and epidemiological analyses. Clin Infect Dis. 2017;64:134–40. 10.1093/cid/ciw691 27988485 PMC5215215

[2] Benedict K, Forsberg K, Gold JAW, Baggs J, Lyman M. *Candida auris*‒Associated Hospitalizations, United States, 2017-2022. Emerg Infect Dis. 2023;29:1485–7. 10.3201/eid2907.230540 37347923 PMC10310363

[3] Morales-López SE, Parra-Giraldo CM, Ceballos-Garzón A, Martínez HP, Rodríguez GJ, Álvarez-Moreno CA, et al. Invasive infections with multidrug-resistant yeast *Candida auris*, Colombia. Emerg Infect Dis. 2017;23:162–4. 10.3201/eid2301.161497 27983941 PMC5176232

[4] Chakrabarti A, Sood P, Rudramurthy SM, Chen S, Kaur H, Capoor M, et al. Incidence, characteristics and outcome of ICU-acquired candidemia in India. Intensive Care Med. 2015;41:285–95. 10.1007/s00134-014-3603-2 25510301

[5] Lyman M, Forsberg K, Sexton DJ, Chow NA, Lockhart SR, Jackson BR, et al. Worsening spread of *Candida auris* in the United States, 2019 to 2021. Ann Intern Med. 2023;176:489–95. 10.7326/M22-3469 36940442 PMC11307313

[6] Centers for Disease Control and Prevention. Tracking *C. auris* [cited 2025 Apr 4]. https://www.cdc.gov/candida-auris/tracking-c-auris/index.html

[7] Carolus H, Sofras D, Boccarella G, Sephton-Clark P, Biriukov V, Cauldron NC, et al. Acquired amphotericin B resistance leads to fitness trade-offs that can be mitigated by compensatory evolution in *Candida auris.* Nat Microbiol. 2024;9:3304–20. 10.1038/s41564-024-01854-z 39567662

[8] Centers for Disease Control and Prevention. Clinical treatment of *C. auris* infections [cited 2025 May 24]. https://www.cdc.gov/candida-auris/hcp/clinical-care/index.html

[9] Lyman M, Forsberg K, Reuben J, Dang T, Free R, Seagle EE, et al. Notes from the field: transmission of pan-resistant and echinocandin-resistant *Candida auris* in health care facilities—Texas and the District of Columbia, January–April 2021. MMWR Morb Mortal Wkly Rep. 2021;70:1022–3. 10.15585/mmwr.mm7029a2 34292928 PMC8297693

[10] Felton T, Troke PF, Hope WW. Tissue penetration of antifungal agents. Clin Microbiol Rev. 2014;27:68–88. 10.1128/CMR.00046-13 24396137 PMC3910906

