# Supplementary material for: Candida auris Testing by the Antimicrobial Resistance Laboratory Network, United States, 2022–2023
Source: Emerg Infect Dis. 2026 Feb;32(2):308–10. doi: 10.3201/eid3202.251043 (PMC12928215; doi:10.3201/eid3202.251043)
Supplement: Appendix — Additional information for Candida auris testing by the Antimicrobial Resistance Laboratory Network, United States, 2022–2023. [file 25-1043-Techapp-s1.pdf]

Article DOI: https://doi.org/10.3201/eid3202.251043

EID cannot ensure accessibility for supplementary materials supplied by authors. Readers who have difficulty accessing supplementary content should contact the authors for assistance.

Candida auris Testing by the Antimicrobial Resistance Laboratory Network, United States, 2022–2023

Appendix

Appendix Table. Antifungal susceptibility testing for clinical *Candida auris* isolates, by region and body site — AR Lab Network, United States, 2022–2023*

	All, no. resistant/no. tested (%)				2022, no. resistant/no. tested (%)				2023, no. resistant/no. tested (%)			
Characteristic	AZO	AMB	ECH	PAN	AZO	AMB	ECH	PAN	AZO	AMB	ECH	PAN
AR Lab Network region† where specimen collected												
Central	18/18 (100)	4/18 (22)	0/18 (0)	0/18 (0)	2/2 (100)	0/2 (0)	0/2 (0)	0/2 (0)	16/16 (100)	4/16 (25)	0/16 (0)	0/16 (0)
Mid-Atlantic	461/469 (98)	290/469 (62)	3/469 (1)	2/469 (0)	141/145 (97)	83/145 (57)	2/145 (1)	1/145 (1)	320/324 (99)	207/324 (64)	1/324 (0)	1/324 (0)
Midwest	666/801 (83)	21/799 (3)	13/799 (2)	0/799 (0)	233/320 (73)	6/320 (2)	6/320 (2)	0/320 (0)	433/481 (90)	15/479 (3)	7/479 (1)	0/479 (0)
Mountain	449/463 (97)	13/463 (3)	14/463 (3)	1/463 (0)	269/276 (97)	2/276 (1)	1/276 (0)	0/276 (0)	180/187 (96)	11/187 (6)	13/187 (7)	1/187 (1)
Northeast	1,418/1,420 (100)	629/1,420 (44)	31/1,420 (2)	10/1,420 (1)	625/626 (100)	121/626 (19)	17/626 (3)	2/626 (0)	793/794 (100)	508/794 (64)	14/794 (2)	8/794 (1)
Southeast	1,488/1,603 (93)	61/1,603 (4)	14/1,603 (1)	1/1,603 (0)	582/646 (90)	47/646 (7)	7/646 (1)	1/646 (0)	906/957 (95)	14/957 (1)	7/957 (1)	0/957 (0)
West	1,915/1,927 (99)	30/1,792 (2)	14/1,908 (1)	1/1,773 (0)	755/762 (99)	12/744 (2)	6/762 (1)	1/744 (0)	1,160/1,165 (100)	18/1,048 (2)	8/1,146 (1)	0/1,029 (0)
Not reported	829/893 (93)	80/894 (9)	8/894 (1)	1/893 (0)	196/201 (98)	19/202 (9)	2/202 (1)	0/201 (0)	633/692 (91)	61/692 (9)	6/692 (1)	1/692 (0)
Body site												
Blood	2,616/2,734 (96)	469/2,696 (17)	14/2,732 (1)	3/2,694 (0)	1,111/1,165 (95)	132/1,164 (11)	7/1,165 (1)	0/1,164 (0)	1,505/1,569 (96)	337/1,532 (22)	7/1,567 (0)	3/1,530 (0)
Respiratory	746/785 (95)	99/761 (13)	3/778 (0)	0/754 (0)	294/316 (93)	28/313 (9)	1/316 (0)	0/313 (0)	452/469 (96)	71/448 (16)	2/462 (0)	0/441 (0)
Urine	2,344/2,477 (95)	354/2,435 (15)	72/2,470 (3)	11/2,426 (0)	846/916 (92)	89/913 (10)	32/917 (3)	5/912 (1)	1,498/1,561 (96)	265/1,522 (17)	40/1,553 (3)	6/1,514 (0)
Wound	843/872 (97)	89/861 (10)	2/870 (0)	0/859 (0)	328/342 (96)	22/342 (6)	1/342 (0)	0/342 (0)	515/530 (97)	67/519 (13)	1/528 (0)	0/517 (0)
Other	695/726 (96)	117/705 (17)	6/724 (1)	2/705 (0)	224/239 (94)	19/229 (8)	0/239 (0)	0/229 (0)	471/487 (97)	98/476 (21)	6/485 (1)	2/476 (0)
Total	7,244/7,594 (95)	1,128/7,458 (15)	97/7,574 (1)	16/7,438 (0)	2,803/2,978 (94)	290/2,961 (10)	41/2,979 (1)	5/2,960 (0)	4,441/4,616 (96)	838/4,497 (19)	56/4,595 (1)	11/4,478 (0)

Abbreviations: AZO = azoles, AMB = amphotericin B, ECH = echinocandins. PAN = pan-resistant, AR Lab Network = Antimicrobial Resistance Laboratory Network

*The tentative minimum inhibitory concentration breakpoints used to define resistance were ≥32 mcg/mL (fluconazole), ≥1.5 mcg/mL (amphotericin B), and ≥4 mcg/mL (echinocandins [anidulafungin or micafungin]).

†https://www.cdc.gov/antimicrobial-resistance-laboratory-networks/php/about/domestic.html.
